# Comparison of Antimicrobial Activity of Injectable Platelet-Rich Fibrin (i-PRF) and Leukocyte and Platelet-Rich Fibrin (l-PRF) Against Oral Microbes: An In Vitro Study

**DOI:** 10.7759/cureus.46196

**Published:** 2023-09-29

**Authors:** Vyshnavi B Sindhusha, Jaiganesh Ramamurthy

**Affiliations:** 1 Periodontics, Saveetha Dental College and Hospitals, Saveetha Institute of Medical and Technical Sciences, Saveetha University, Chennai, IND; 2 Periodontics, Saveetha Dental College and Hospitals, Saveetha institute of medical and technical sciences, Saveetha University, Chennai, IND

**Keywords:** leukocyte and platelet rich fibrin (l-prf), injectable platelet rich fibrin (i-prf), oral microbes, porphyromonas gingivalis, antibacterial activity

## Abstract

Aim

Injectable platelet-rich fibrin (i-PRF) and leukocyte and platelet-rich fibrin (l-PRF) are both blood-derived products used in regenerative medicine and dentistry. They contain platelets, growth factors, and leukocytes, which can have antimicrobial properties to some extent, but their primary purpose is tissue regeneration and wound healing. i-PRF and l-PRF may have some indirect antimicrobial properties due to their composition and ability to enhance tissue healing and immune responses, and they are primarily used in dentistry for their regenerative and wound healing capabilities rather than as standalone antimicrobial agents.

This study aims to compare the anti-microbial activity of i-PRF and l-PRF against oral microbes.

Methodology

This study included 30 patients who were selected using G*Power software version 3.1 (Heinrich-Heine-Universität Düsseldorf, Düsseldorf, Germany) calculation with the population size. The plaque samples were collected from the subjects using area-specific Gracey curettes used for scaling and root planing to remove plaque and calculus from the teeth and root surfaces. The collected plaque samples were transferred to a tube containing 5 ml of saline (sterile saltwater). The purpose of using saline is to preserve the microbial content of the plaque sample without altering the microbial composition. To obtain a uniform solution, the samples in the saline-containing tube were vortexed for 5 minutes. After vertexing, a small amount of the suspension (0.1 ml) was taken for further analysis. The 0.1 ml suspension was used to plate blood agar using the streak method. A loop or needle is used to streak the sample back and forth across the surface of the agar, leading to the dilution and separation of the bacteria.

Results

Results state that i-PRF has a maximum zone of inhibition (2.19±0.47 mm) when compared with metronidazole (0.14±0.09 mm). It can be stated that platelet concentrates demonstrate better antimicrobial activity due to their higher oxygen metabolites which help in the aggregation and internalization of microorganisms, which enhances the clearance of pathogens from the bloodstream. Paired t-test has been used for the comparison between the two groups, and the p-value is >0.05 stating that the difference is statistically significant.

Conclusion

The present study states that i-PRF demonstrated better antimicrobial efficacy as compared to l-PRF. Hence, i-PRF helps in reducing microbial load at the periodontally infected sites when compared with l-PRF.

## Introduction

Dental plaque, also known as dental biofilms, refers to a complex community of microorganisms that adhere to each other and/or dental surfaces or interfaces within the oral cavity. These biofilms are encased in a matrix composed of extracellular polymeric substances (EPS), which are mainly produced by the microorganisms themselves. The EPS matrix provides protection and stability to the microbial community, making dental biofilms resistant to mechanical removal and antimicrobial agents [1]. In deep periodontal pockets, which are the spaces between the teeth and gums that have become deeper due to periodontal disease, biofilm development is a significant factor contributing to disease progression. Studies on biofilm formation in these deep pockets have shown distinct patterns of colonization. The deepest sites of periodontal pockets tend to be colonized predominantly by motile species of bacteria. The red complex is a group of three specific bacterial species commonly found in periodontal pockets: *Porphyromonas gingivalis *(Pg), *Treponema denticola*, and *Tannerella forsythia*. These bacteria are considered major periodontal pathogens and are associated with severe periodontitis [2]. The composition of dental biofilms can vary depending on the location within the oral cavity. In shallow sites, such as the enamel or root surface, a different microbial composition is often observed compared to deep periodontal pockets.

Non-surgical periodontal therapy, also known as scaling and root planing or deep cleaning, is the initial treatment approach for managing periodontal disease. The main goals of this therapy are to eliminate the microbial and inflammatory factors contributing to the disease and to reduce the depth of periodontal pockets to a more manageable level. The desired outcome is typically achieving pocket depths of 5 millimeters or less. Different antimicrobial agents are used as adjuncts to the non-surgical periodontal therapy. If the pocket depths are not reduced to 5 mm, then surgical periodontal therapy will be performed.

Tissue-engineering techniques have shown promise in regenerative procedures following non-surgical periodontal therapy or for bone augmentation in dental and oral surgery. One such technique is the use of leukocyte and platelet-rich fibrin (l-PRF), which is a second-generation platelet concentrate and an autologous biomaterial. l-PRF is prepared from the patient's own blood, making it an autologous product. l-PRF concentrates a high percentage of platelets (>90%) and leukocytes (>75%) the source of the blood [3]. Platelets are rich sources of growth factors that play a crucial role in tissue regeneration, while leukocytes are essential for immune response and tissue healing. l-PRF serves as a scaffold that supports the regenerative cells in the treated area. When placed at a surgical site, l-PRF provides a three-dimensional matrix that helps in the proliferation and differentiation of cells involved in regeneration [4]. l-PRF offers a sustained release of growth factors and other bioactive substances. l-PRF can be prepared in various forms, with the membrane being the most commonly used. Other forms include plugs and liquid formulations, which can be adapted for specific regenerative needs [5].

Injectable platelet-rich fibrin (i-PRF) is a relatively recent advancement in the field of platelet concentrates. It is a form of PRF that is used in an injectable form, and it coagulates within a few minutes after being injected at the treatment site. i-PRF is prepared in a way that allows it to be administered via injection. After injection, i-PRF undergoes coagulation, forming a fibrin gel or clot. The coagulation process helps in creating a stable and localized environment for cell migration, proliferation, and tissue regeneration [6]. i-PRF goes beyond standard PRF by also containing stem cells and endothelial cells. These additional cell types have regenerative potential and can contribute to tissue repair and angiogenesis (formation of new blood vessels). i-PRF is also an autologous product and releases growth factors and bioactive substances over time, promoting tissue repair and regeneration at the injection site [7].

Since i-PRF and l-PRF may have some indirect antimicrobial properties due to their composition along with their ability to enhance tissue healing and immune responses, these are primarily used in dentistry for their regenerative and wound healing capabilities rather than as antimicrobial agents. Hence, this study aims to compare the anti-microbial activity of i-PRF and l-PRF against oral microbes.

## Materials and methods

Inclusion criteria

**Figure 1:**
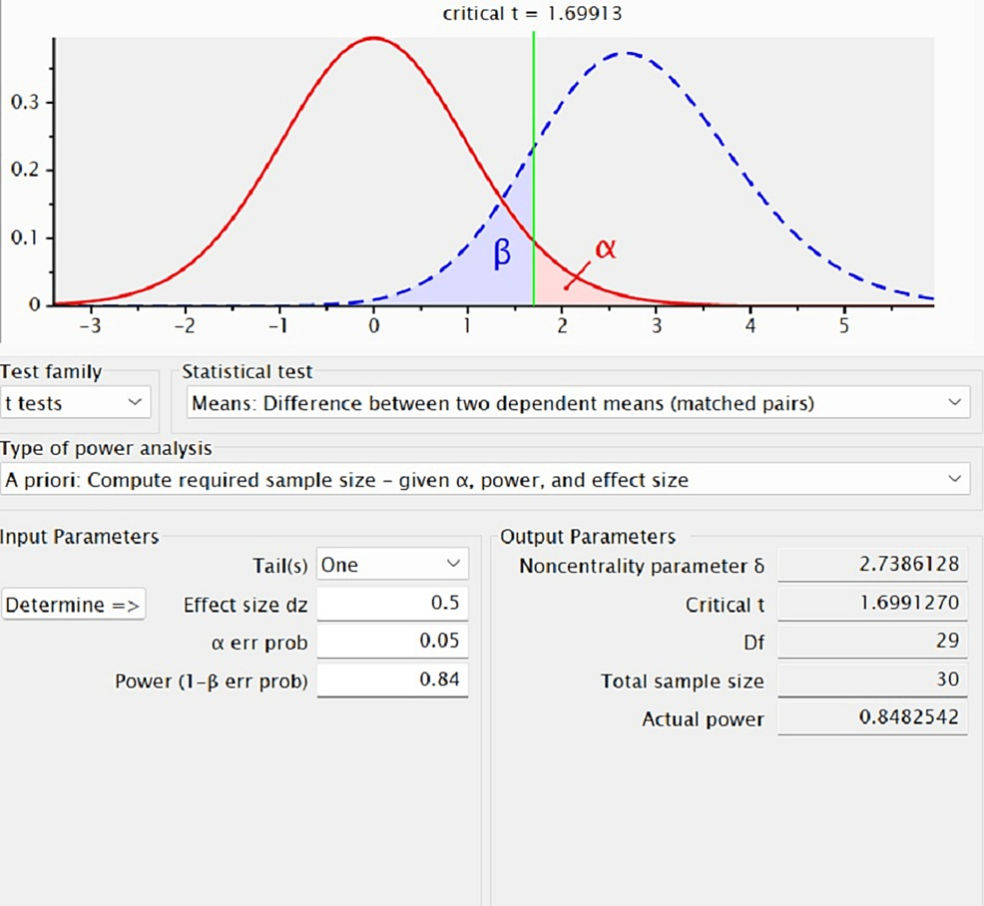
The sample size calculation was done using G*Power software version 3.1 calculation

The participants for the study were selected from individuals visiting the Department of Periodontics at Saveetha Dental College and Hospitals. The participants were informed about the study conducted, and informed consent was taken from each participant. The study focused on individuals who were both systemically and periodontally healthy. The samples collected from the participants were analyzed for their antimicrobial activity, and analysis was conducted. Before initiating the research, ethical clearance was obtained to ensure that the study complies with ethical guidelines and protects the rights and well-being of the study participants. A total of 30 individuals above 25 years of age were included in this study (n=30). The selection criteria required these participants to be systemically and periodontally healthy. The gingival index was recorded in the participants. The sample size calculation was done using G*Power software version 3.1 (Heinrich-Heine-Universität Düsseldorf, Düsseldorf, Germany) based on the population size (Figure 1).

Exclusion criteria

Individuals with a probing depth of 3 mm or more along with clinical attachment loss were excluded. Individuals who have a habit of smoking or using tobacco products were excluded. Individuals with any systemic diseases or infections were excluded. Individuals who have been using anti-inflammatory or antibiotic drugs for the last six months were excluded. Pregnant women and lactating mothers were also excluded from the study.

Study design

Before participating in the study, the participant's informed consent was taken. A total of 10 ml of blood was taken from each participant. Blood samples were collected to prepare both i-PRF and l-PRF. For i-PRF preparation, 5 ml of blood was placed into a blood-collection tube without any additives. The tube containing the blood was then centrifuged at 700 rpm for 3 minutes. For l-PRF preparation, another 5 ml of blood was drawn into a glass collection tube without any additives. The tube containing the blood was then centrifuged at 3000 rpm for 10 minutes. The higher speed and longer duration of centrifugation in the l-PRF preparation process are likely used to obtain a different concentration of platelets and other cellular components.

**Figure 2:**
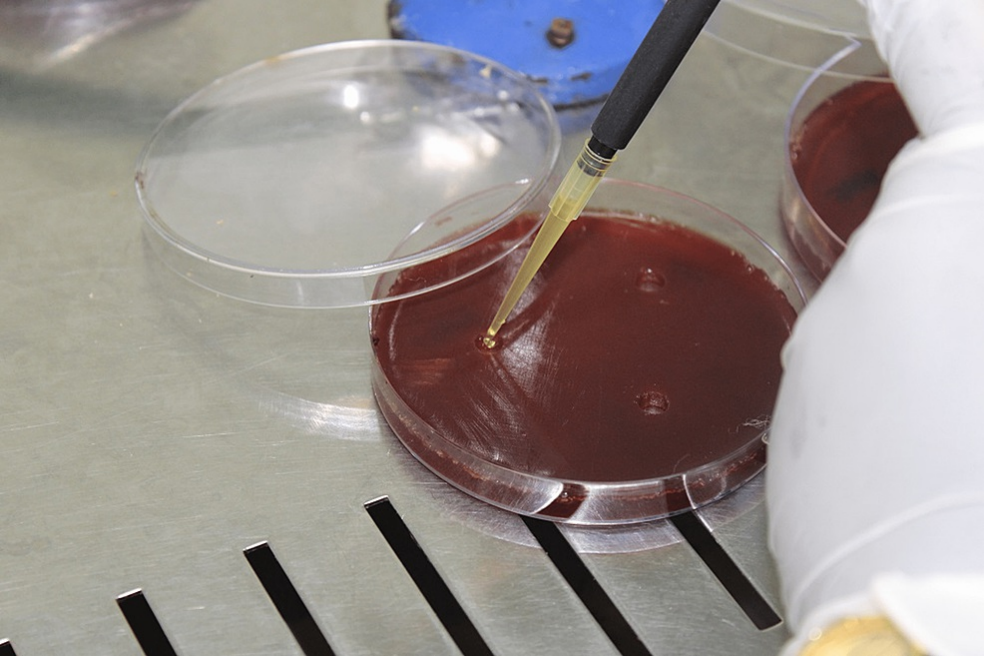
The agar plates were inoculated with the i-PRF and l-PRF The agar plates were inoculated with i-PRF and l-PRF and placed in the anaerobic environment for culture.

Agar plates containing the bacterial strains Pg and Aa were prepared and inoculated. These plates provide a suitable medium for the growth of the bacteria. On each plate, a marker was used to identify the location of Pg colonies. The l-PRF, i-PRF, and metronidazole samples were divided into three compartments. Each compartment was placed around the identified Pg colonies on the agar plates (Figure 2).

After sample placement, the agar plates were incubated for 24 hours in an anaerobic atmosphere at a temperature of 37°C. After incubation, the researchers measured the zone of inhibition surrounding the samples on the agar plates. The size of the clear zone of inhibition is used to evaluate the antibacterial efficacy of l-PRF, i-PRF, and metronidazole against Pg. The agar plates showed the zones of inhibition around the samples.

## Results

**Figure 3:**
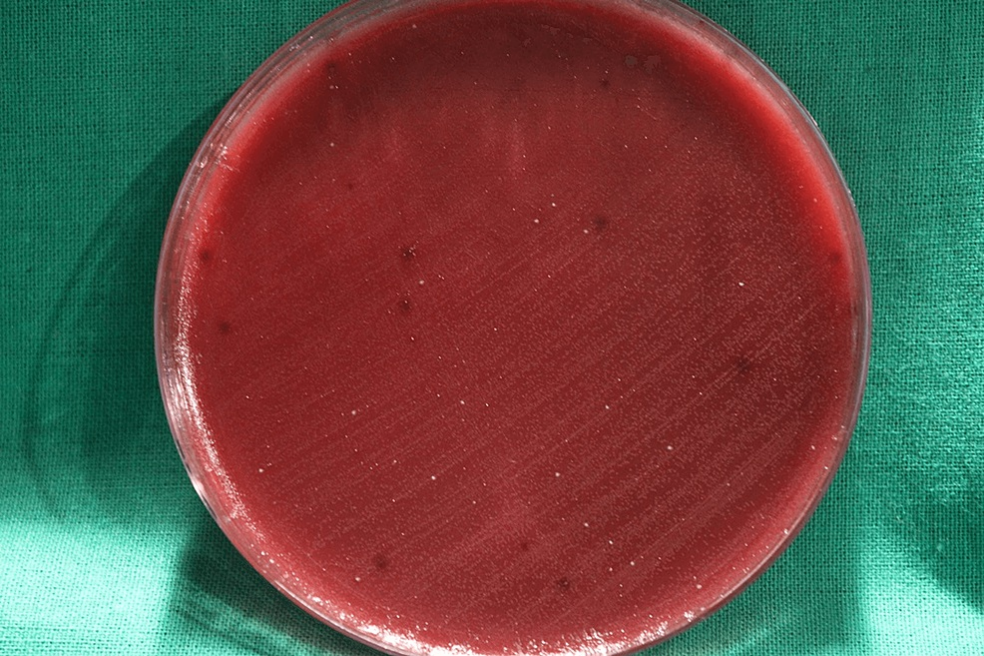
Black-pigmented colonies Black-pigmented colonies showing the Pg, when the i-PRF and metronidazole were applied on the agar plate using a streak method

After incubation of 24 hours, a greater zone of inhibition was seen in the platelet region (i-PRF and l-PRF) on the agar plates inoculated with Pg. These zones indicate the areas where the growth of Pg was inhibited due to the antimicrobial properties of the platelet concentrates as seen in Figure 3.

The mean along with the standard deviation for the widths of the zones of inhibition for i-PRF and l-PRF against Pg were measured, and the corresponding standard deviations are listed in Table 1.

**Table 1:** The zone of inhibition of the different platelet concentrates i-PRF: injectable platelet-rich fibrin, l-PRF: leukocyte and platelet-rich fibrin

Platelet concentrate	Zone of inhibition (mm)	Standard deviation (mm)
i-PRF	2.19	0.47
l-PRF	1.32	0.30
Metronidazole	0.14	0.09

Table 1 shows the average size of the zones of inhibition for i-PRF, l-PRF, and metronidazole against Pg. In the case of Pg, I-PRF demonstrated the widest zone of inhibition, and this difference was found to be statistically significant when compared to PRF. Furthermore, i-PRF exhibited a significantly wider zone of inhibition compared to l-PRF. A paired t-test was used to compare the significance between the two study groups, and the difference between i-PRF and l-PRF was not statistically significant with a p-value of >0.05.

## Discussion

Platelet concentrates have gained significant attention and usage in periodontal surgeries due to their regenerative potential and ability to enhance wound healing. Platelets, a type of blood cell, are rich in growth factors, which are bioactive proteins that play a crucial role in tissue repair and regeneration. When activated, platelets release several growth factors, including platelet-derived growth factor (PDGF), transforming growth factor beta (TGF-β), vascular endothelial growth factor (VEGF), and insulin-like growth factor (IGF). Platelets also contribute to tissue regeneration by providing a temporary matrix for connective tissue formation. Upon activation, they secrete proteins such as fibrin, fibronectin, and vitronectin, which help create a scaffold for cell migration and support tissue repair [8]. Various techniques have been developed to prepare platelet concentrates for clinical use. Some of the commonly used platelet concentrate preparations include platelet-rich plasma (PRP), platelet-rich fibrin (PRF), and platelet-rich growth factor (PRGF). The use of platelet concentrates in periodontal surgeries aims to accelerate healing, promote tissue regeneration, and enhance the overall outcome of the procedures [9].

Autologous platelet concentrates, such as PRP and PRF, are used in regenerative medicine and periodontal surgeries to reduce the risk of infection and antibody production against foreign substances. There are some concerns associated with the use of certain additives during platelet concentrate preparation, such as thrombin and calcium. Thrombin is a clotting factor that plays a key role in converting fibrinogen into fibrin, leading to clot formation. It is often used in PRP preparation to initiate the clotting process and form a fibrin gel, and it may also lead to antibody production against thrombin and other clotting factors, such as V and XI. These antibodies can have negative implications for the coagulation process and may trigger an immune response, leading to potential complications [10].

PRF offers an alternative approach that avoids the use of anticoagulants or bovine thrombin. PRF is generated by centrifuging blood without any additives, and the resulting product contains a fibrin clot with a concentrated mixture of platelets, leukocytes, and growth factors. By eliminating the need for thrombin and anticoagulants, PRF reduces the risk of immunological responses and adverse reactions associated with these additives. The use of PRF has gained popularity in various medical and dental fields due to its effectiveness in promoting tissue regeneration and wound healing [11].

In PRF preparation, the absence of anticoagulants and bovine thrombin allows the natural polymerization of fibrinogen to fibrin using the physiological levels of thrombin present in the patient's blood. This slow and natural polymerization process leads to the formation of a three-dimensional fibrin matrix that encapsulates a concentrated mixture of platelets, leukocytes, and growth factors [12]. The architecture of the fibrin matrix provides a scaffold or matrix that supports tissue regeneration and prevents the rapid proteolysis (breakdown) of growth factors. As the fibrin matrix gradually disintegrates over time (usually within 7-11 days), the growth factors and cytokines encapsulated within it are progressively released into the surrounding tissues. This sustained release of growth factors is beneficial as it promotes cell proliferation, collagen synthesis, and other regenerative processes over an extended period [13].

Another key advantage of PRF is its ability to enhance micro vascularization which refers to the formation of new small blood vessels (capillaries) in the vicinity of the wound. These new blood vessels play a vital role in supplying oxygen, nutrients, and immune cells to the regenerating tissues, which aids in the healing process. The slow degradation of the fibrin matrix in PRF, along with the gradual release of growth factors, provides a favorable environment for tissue repair and regeneration [14]. i-PRF is a modification of the traditional PRF concept that offers the advantage of being in a liquid or gel-like form, making it easier to use in various clinical applications. i-PRF is obtained by modifying the centrifugation parameters during preparation, leading to a slower and shorter spin compared to standard PRF [15].

The key advantages of i-PRF are its injectable form, which has a greater number of regenerative cells, greater concentration of growth factors, and sustained release of growth factors. The injectable nature of I-PRF can be used as a stand-alone treatment or in combination with other biomaterials, such as bone grafts or scaffolds, to enhance their regenerative properties. Several studies have investigated the antibacterial properties of different platelet concentrates against specific periodontal pathogens [16].

Yang et al. (2015) compared the antimicrobial activity of four plasma fractions, including PRP and PRF, against periodontal pathogens such as Pg, *Aggregatibacter actinomycetemcomitans* (Aa), and *Fusobacterium nucleatum* [17]. They found that PRP exhibited the highest antibacterial activity among the tested fractions. The study done by Karde et al. (2017) evaluated the antibacterial activity of PRP, PRF, and i-PRF against supragingival plaque. i-PRF showed the maximum zone of inhibition, followed by PRP and then PRF [18]. Overall, the results from these studies suggest that PRP tends to have higher antimicrobial activity compared to PRF and i-PRF, especially against specific periodontal pathogens. The antimicrobial efficacy can be influenced by various factors, including the preparation method of the platelet concentrate, the concentration of growth factors, and the target bacterial species. Additionally, the concentration of platelets and leukocytes in the platelet concentrate may also play a role in determining its antimicrobial properties [19].

The higher antimicrobial activity of I-PRF, especially against specific bacteria like Pg, can be attributed to its greater concentration of platelets and other blood cells, such as leukocytes. The "low-speed concept" proposed by Ghanaati et al. suggests that lower centrifugation speeds can result in higher concentrations of cells, including leukocytes, in the final platelet concentration before the formation of the fibrin clot [20]. This indicates that I-PRF, with its modified centrifugation parameters, might retain a higher number of platelets and cells, potentially contributing to its enhanced antimicrobial properties.

In a study done by Joshi et al., Aa showed smaller inhibitory zones when exposed to i-PRF compared to l-PRF [21]. This suggests that Aa may be more resistant to the platelets, leukocytes, and other components present in higher quantities in I-PRF. Additionally, the presence of sodium citrate in PRP, which has known antibacterial properties, might have contributed to the antibacterial effect against Aa. Studying I-PRF's antibacterial activity over an extended period can help understand its duration of effect and whether it maintains higher antimicrobial efficacy compared to other platelet concentrates [22].

i-PRF contains a complex mixture of platelets, white blood cells (leukocytes), growth factors, and plasma components. They release antimicrobial peptides and myeloperoxidase that help in activating specific antigen-antibody responses along with the antioxidants. As mentioned, platelet concentrates contain leukocytes and platelets in significantly higher concentrations than whole blood. Leukocytes, especially neutrophils and monocytes, play a vital role in the immune response and possess antimicrobial actions. Monocytes are involved in producing cytokines and chemotactic substances, which play roles in inflammation and recruiting immune cells to the site of infection [23].

Platelet microbicidal proteins (PMPs) are a group of compounds found in platelet concentrates that exhibit antibacterial activity. These PMPs, including fibrinopeptide B, platelet factor 4, connective tissue activating peptide 3, thymosin beta 4, platelet basic protein, and platelet factor 4, can interact with bacterial membranes, alter their permeability, penetrate the bacterial cells, and inhibit the synthesis of critical components, ultimately leading to bacterial destruction. i-PRF and l-PRF, being entirely autologous and exhibiting antibacterial activity, contribute to improved wound healing and periodontal regeneration while potentially reducing the need for high antibiotic doses. Thus, the antimicrobial properties of platelet concentrates and their integration into clinical practice may offer novel and effective strategies for managing periodontal infections and promoting optimal patient outcomes [24].

Limitations

Antimicrobial activity of i-PRF and l-PRF against black pigment organisms should be established against a larger sample size. Further research is required related to the i-PRF and l-PRF antimicrobial activity. Using this study, we are able to say that the antimicrobial property of i-PRF and l-PRF is above and equal to the antimicrobial activity of metronidazole against periodontal pathogens.

## Conclusions

The two platelet concentrates showed antimicrobial activity against Pg, in which i-PRF was proven to be the most powerful against the black-pigmented organisms when it was compared against metronidazole. l-PRF showed equal antimicrobial activity as metronidazole. However, i-PRF and l-PRF have additional use as they are completely autologous without additives, and along with the above advantages, the I-PRF application is also the least invasive.
